# Treatment outcomes of patients with multidrug and extensively drug-resistant tuberculosis in Zhejiang, China

**DOI:** 10.1186/s40001-021-00502-0

**Published:** 2021-04-03

**Authors:** Ming-Wu Zhang, Lin Zhou, Yu Zhang, Bin Chen, Ying Peng, Fei Wang, Zheng-Wei Liu, Xiao-Meng Wang, Song-Hua Chen

**Affiliations:** 1 Zhejiang Provincial Center for Disease Prevention and Control, Hangzhou, Zhejiang China; 2 Zhejiang Public Health Research Institute, Zhejiang, China

**Keywords:** Prognostic factors, M/XDR-TB treatment, Treatment failure, Smear-negative, Adverse effects

## Abstract

**Background:**

The aim of this study was to assess the treatment outcomes of multidrug and extensively drug-resistant tuberculosis (M/XDR-TB) in Zhejiang, China and to evaluate possible risk factors associated with poor outcomes of M/XDR-TB.

**Methods:**

Two-hundred-and-sixty-two patients having M/XDR-TB who received the diagnosis and treatment at nine referral hospitals from 1 January 2016 to 31 December 2016 in Zhejiang, China were included. All patients received second-line regimens recommended by WHO under the DOTS-Plus strategy.

**Results:**

Among the 262 patients, the treatment success rate was 55.34% (*n* = 145) with 53.44% (*n* = 140) cured and 1.91% (*n* = 5) who completed treatment, 62 (23.66%) failed, 27 (10.31%) died, 16 (6.11%) defaulted and 12 (4.58%) transferred out. Forty (64.52%) of the 62 M/XDR-TB patients who failed treatment were due to adverse effects in the first 10 months of treatment. Eighteen patients (6.37%) had XDR-TB. Treatment failure was significantly higher among patients with XDR-TB at 50% than that among patients with non-XDR-TB at 21.72% (*P* = 0.006). Failure outcomes were associated with a baseline weight less than 50 kg (OR, 8.668; 95% CI 1.679–44.756; *P* = 0.010), age older than 60 years (OR, 9.053; 95% CI 1.606–51.027; *P* = 0.013), hemoptysis (OR, 8.928; 95% CI 1.048–76.923; *P* = 0.045), presence of cavitary diseases (OR, 10.204; 95% CI 2.032–52.631; *P* = 0.005), or treatment irregularity (OR, 47.619; 95% CI 5.025–500; *P* = 0.001).

**Conclusion:**

Treatment outcomes for M/XDR-TB under the DOTS-Plus strategy in Zhejiang, China were favorable but still not ideal. Low body weight (< 50 kg), old age (> 60 years), severe symptoms of TB including cavitary disease, hemoptysis and irregular treatment were independent prognostic factors for failure outcomes in patients with M/XDR-TB.

**Supplementary Information:**

The online version contains supplementary material available at 10.1186/s40001-021-00502-0.

## Introduction

Drug-resistant tuberculosis (TB) remains a growing threat to public health and there were an estimated 1.2 million TB deaths among human immunodeficiency virus (HIV) -negative people and an additional 208,000 deaths among HIV-positive people in 2019 [1]. About half a million people developed rifampicin-resistant TB (RR-TB) worldwide in 2019 [2], of which 78% had multidrug-resistant TB (MDR-TB) [3]. A global total of 206 030 people with MDR/RR-TB were detected and almost 50% of the MDR-TB cases worldwide are estimated to occur in China and India [1]. Extensively drug-resistant tuberculosis (XDR-TB) is defined as MDR-TB with resistance to fluoroquinolone and at least one second-line injectable agent (i.e. amikacin, kanamycin, and/or capreomycin). XDR-TB has been reported from more than 58 countries and is estimated to occur in up to 10% of MDR-TB patients [3].

A five-year study revealed that, of 9544 MTB isolates, there were 3376 (35.4%), 842 (8.8%) and 61 (0.64%) isolates identified as MDR-TB, XDR-TB and XDR-TB-Plus, respectively. The proportion of XDR-TB showed significant increase from 6.3% in 2011 to 9.1% in 2015[4].In Zhejiang province China, MDR-TB showed a decreasing trend, while resistance to any first-line drugs showed an increasing trend, and 3.21%) and 21.28% MDR-TB cases were registered as new and previously treated cases, respectively. [5]. The prevalence of M/XDR-TB in Zhejiang Province underscores the continued need for effective treatment programs for drug-resistant TB.

This study aimed to investigate the outcomes of M/XDR-TB patients who were previously treated at provincial TB referral hospitals using the “DOTS-Plus” (a complementary DOTS-based strategy with provisions for treating multidrug-resistant tuberculosis) strategy, and to identify possible risk factors associated with poor treatment outcomes.

## Methods

### Study area, participants and procedures

The study area consisted of seven cities (Hangzhou, Huzhou, Shaoxing, Lishui, Quzhou, Jiaxing, and Wenzhou) including 39 counties with an urban area of about 19.2 million people in Zhejiang Province, China.

A total of 262 patients with culture-confirmed M/XDR-TB who accepted treatment at the 9 referral hospitals from 1 Jan 2016 to 31 Dec 2016 including 18 XDR-TB patients were included in this study.

The definitions of MDR-TB and XDR-TB as defined by the WHO were used in this study. Patients diagnosed with M/XDR-TB based on the drug susceptibility test (DST) results received a standardized or individual treatment regimen (WHO) [6].

Standard definitions for MDR-TB treatment outcome as defined by the suggested criteria of J.E. Farley, M. Ram et al*.* were used in this project [7]. A patient was considered successfully treated if consistently culture-negative for the final 12 months of treatment with completed treatment which is defined as “cured” or if unknown for bacteriological results but does not meet the definition for cure which is defined as “treatment completed”. An MDR-TB patient treatment interrupted for 2 or more consecutive months for any reason was defined as treatment default, while patient transferred to another unit with unknown treatment outcome was classified as “transfer out”. “Failure” is regarded as 2–5 positive cultures or 1 of any 3 positive cultures during the final 12 month of treatment.

### Data collection

The treatment record of each patient was collected, e.g. information about symptoms of TB, baseline weight, and other characteristics (Table1). Results from the microbiology laboratory performed at the time of diagnosis of M/XDR-TB were reviewed and analyzed. All smear microscopy and culture on Lowenstein–Jensen media were positive. For all cases, isolates were sent for DST using the procedures of the provincial reference laboratories. All patients with M/XDR-TB isolates obtained prior to starting DOTS-Plus were sent to DST when the isolates were resistant to INH at 1 ug/ml and RMP at 40 ug/ml. In addition, these isolates sent to the laboratory for DST were tested for (resistance concentration): ethambutol (E), 2 ug/ml; kanamycin (Km), 30 ug/ml; streptomycin (S), 4 ug/ml; and ofloxacin (Ofx), 2 ug/ml.

**Table 1:** Characteristic of 262 patients with M/XDR-TB enrolled in the DOTS-Plus program in Zhejiang, China between 1 January and 31 December 2016

Characteristic	Total M/XDR-TB	XDR-TB	Non-XDR-TB	^χ2^	*P*
	(*n* = 262)	*(n* = 18)	(*n* = 244)		
Sex					
Male	192(73.28)	13	179	0.011	0.916
Female	70(26.72)	5	65		
Age					
< 45	128(48.85)	5	123	4.776	0.092
45–59	69(26.34)	5	64		
≥ 60	65(24.81)	8	57		
Occupation^a^					
Farmer	147(56.11)	10	137	2.054	0.152
Others	115(43.89)	8	107		
Family register^b^					
Resident	124(47.33)	8	116	0.064	0.800
Floating	138(52.67)	10	128		
Previous TB treatment					
No	21(8.02)	0	21	1.684	0.194
Yes	241(91.98)	18	223		
TB symptoms					
Hemoptysis	181(69.08)	13	168	0.089	0.765
Cavity	127(48.47)	13	114	6.752	0.009
Hospitalized	66(25.19)	2	64	2.033	0.154
Comorbidities					
Impaired renal function	82(31.30)	6	76	3.795	0.164
Diabetes	5(1.91)	0	5	3.433	0.150
Liver disease	2(0.76)	0	2	3.620	0.150

Data are No. (%)

^a^Others including students and other occupations not investigated specifically

^b^Permanent local residents were classified as “resident”, others as “Floating”

### Treatment of patients with M/XDR-TB

All cases were in residence in an area with DOTS-Plus implementation approved by the Green Light Committee (GLC) according to the Guidelines for the Programmatic Management of Drug-Resistant Tuberculosis [8]. Two-hundred-and-thirty-five (89.69%) M/XDR-TB patients were treated under a standard regimen using the treatment protocol of M/XDR-TB and treatments of 27 (10.31%) were individualized by each referral hospital on the basis of DST results and adverse reactions. An injectable agent (including aminoglycoside or capreomycin) was used for a minimum of 6 months and at least 4 months past culture conversion. After the initial intensive treatment, the whole duration was 24 months.

### Statistical analysis

The data were checked for completeness and consistency. Crude odds ratios (OR) and 95% confidence intervals (CI) were calculated using a stepwise logistic regression analysis with SPSS version 17.0 (SPSS, Inc., Chicago, IL, USA). The χ^2^ or the Fisher’s exact test was used to determine the significant differences in frequencies of values in various groups where *P* < 0.05 was considered significant.

## Results

### Patients’ characteristics

In this study, 262 cases of M/XDR-TB were recruited for treatment, with 18 (6.87%) having XDR-TB. The median age of the patients with XDR-TB and Non-XDR-TB were 52.83 ± 14.95 years (range 29–77) and 46.07 ± 16.17 years (range 10–81), respectively. The majority of the patients (73.28%, *n* = 192) were male. The baseline characteristics of tuberculosis patients are shown in Table 1.

There was no significant difference between patients infected with MDR-TB and XDR-TB in terms of baseline weight, comorbidities, and laboratory findings. However, the presence of cavities at the time of diagnosis was more common in XDR-TB patients than those with Non-XDR-TB (72.22% vs 46.72%**,** *P* = 0.009) (Table 1).

**Fig. 1:**
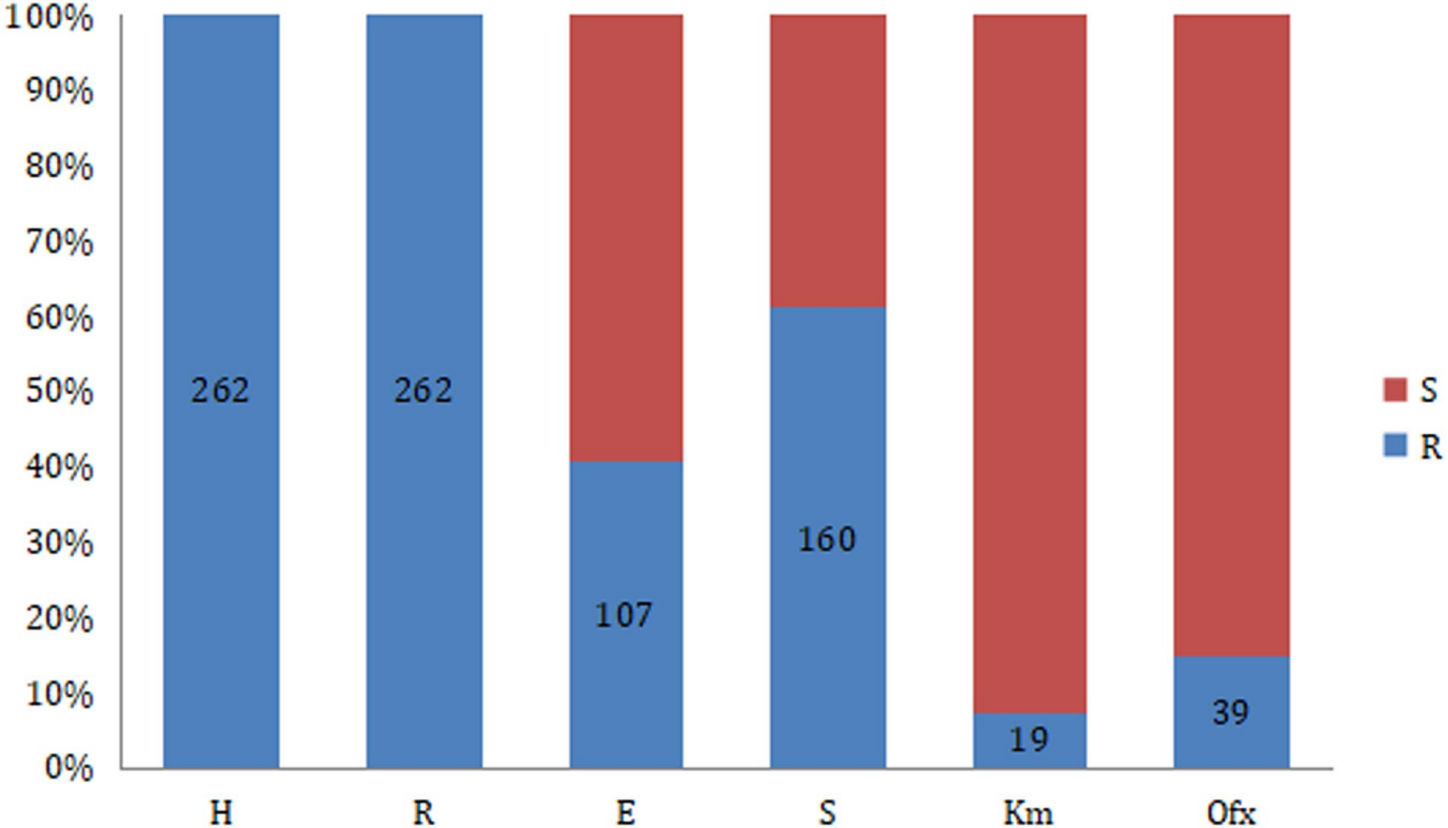
Histogram of resistance and sensitivity for individual drugs, “resistant (R)” is marked in blue and “sensitive (S)” is marked in red

**Fig. 2:**
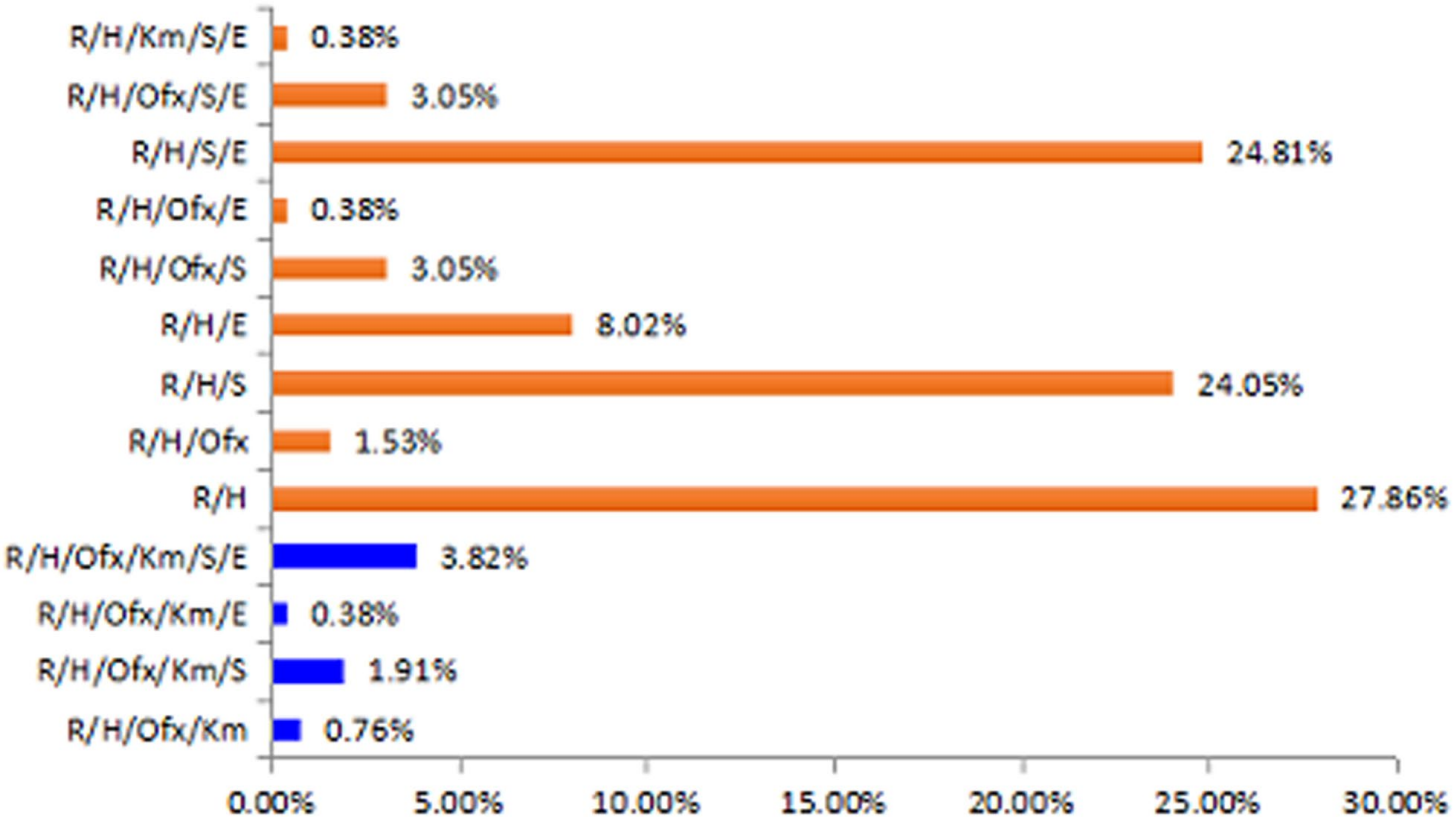
Percentage of resistance to each combination of drugs among the isolates from the 262 patients. XDR-TB is marked in blue and MDR-TB is marked in red

The resistance patterns are shown in Fig. 1; all patients were resistant to isoniazid and rifampicin. Among the second-line drugs, resistance to streptomycin (160, 61.07%) and ethambutol (107, 40.84%) was common; while resistance to ofloxacin (39, 14.89%) and kanamycin (19, 7.25%) was rare. Overall, 33.6% of the strains from the patients were resistant to at least one second-line anti-TB drug (ethambutol, streptomycin or ofloxacin). Multidrug resistance patterns were predominantly resistant to R/H, R/H/S /E and R/H/S at 27.86%, 24.81% and 24.05%, respectively (Fig. 2). Resistance to 5 or more drugs accounted for 9.54% of the multidrug resistance.

### Outcomes of the treatment

Among all patients, the treatment success rate was 55.34% (*n* = 145) with 53.44% (*n* = 140) cured and 1.91% (*n* = 5) treatment completed. Treatment failure occurred in 62 patients (23.66%) and 27 (10.31%) died during treatment. A further 16 (6.11%) patients defaulted and 12 (4.58%) patients transferred out to other cities with unknown treatment outcome (Table 2). Of 140 cured patients, 136 (97%) converted to smear-negative in the first 6 months of treatment (χ^2^ = 113.540, *P* < 0.001). Sixteen deaths (59.26% of the deaths) occurred in the first 6 months and 11 (40.74%) additional deaths occurred during the remaining 18 months of the treatment program.

**Table 2:** Treatment outcomes and adverse reactions among patients with M/XDR-TB

Treatment outcome	Total MDR-TB	XDR-TB	Non-XDR-TB	^χ2^	*P*
	(*N* = 262), *n *(%)	(*N* = 18), *n* (%)	(*N = *244), *n* (%)		
Treatment success (Cure, TC)	145(55.34)	6(33.33)	139(56.97)	3.789	0.052
Cure	140(53.44)	6(33.33)	134(54.92)	2.331	0.126
Completed	5(1.91)	0(0.00)	5(2.05)	0.078	0.779
Failure	62(23.66)	9(50.00)	53(21.72)	7.420	0.006
Death	27(10.31)	2(11.11)	25(10.25)	0.081	0.775
Default	16(6.11)	0(0.00)	16(6.56)	0.373	0.541
Transfer out	12(4.58)	1(5.56)	11(4.51)	0.143	0.704
Adverse drug reactions	122 (46.55)	7(38.89)	115(47.13)	0.458	0.499
GI upset^a^	98(37.40)	5(27.78)	93(38.11)	2.313	0.315
Hepatotoxicity	36(13.74)	1(5.56)	35(14.34)	5.522	0.063
Allergic reaction	17(6.49)	0(0.00)	17(6.97)	6.281	0.043
Neurologic abnormalities	5(1.91)	0(0.00)	5(2.05)	5.434	0.066
Mental disorder	2(0.76)	0(0.00)	2(0.82)	5.296	0.071
Hematologic abnormalities	2(0.76)	0(0.00)	2(0.82)	5.296	0.071
Electrolyte disturbances	3(1.15)	0(0.00)	3(1.23)	5.429	0.068
Renal toxicity	5(1.91)	0(0.00)	5(2.05)	5.434	0.066
Arthralgia or Courbature	25(9.54)	2(11.11)	23(9.43)	3.975	0.137
Hypothyroidism	10(3.82)	1(5.56)	9(3.69)	5.049	0.080

^a^*GI* gastrointestinal

The median duration of treatment success was 730.60 days (95% CI 717–731 days) for patients with XDR-TB and 730.65 days (95% CI 730.48–730.80 days) for patients with Non-XDR-TB.

### Risk factors for treatment failure

The difference in treatment outcome was not statistically significant for gender, occupation, family register or other characteristics (Table 3).

**Table 3:** Factors associated with Treatment failure of 262 patients with M/XDR-TB

Chracteristic	Treatment success^c^		Treatment failure		Total		Univariate analysis		Multivariate analysis	
	Cure, TC *n*(%)		Failure (*n*,%)		*N* (%)		OR (95% CI)^d^	*P*	OR (95% CI)	*P*
Sex										
Male	105	54.69	42	21.88	192	73.28	1.25(0.656–2.383)	0.498	0.66(0.109–4.011)	0.652
Female	40	57.14	20	28.57	70	26.72	1		1	
Age										
< 60 year	124	62.94	35	17.77	197	75.19	4.555(2.302–9.015)	0.000	9.053(1.606–51.027)	0.013
≥ 60 year	21	32.31	27	41.54	65	24.81	1		1	
Occupation^a^										
Farmer	108	54.82	47	23.86	197	75.19	0.932(0.467–1.859)	0.841	1.343(0.262–6.898)	0.724
Others	37	56.92	15	23.08	65	24.81	1		1	
Family register^b^										
Resident	62	50	38	30.65	124	47.33	0.472(0.257–0.866)	0.015	5.502(0.778–38.935)	0.088
Floating	83	60.14	24	17.39	138	52.67	1		1	
Previous TB treatment										
No	14	66.67	4	19.05	21	8.02	1.55(0.489–4.911)	0.457	2.033(0.113–36.662)	0.631
Yes	131	54.36	58	24.07	241	91.98	1		1	
Weight										
< 50 kg	19	44.19	16	37.21	43	16.41	2.824(1.198–6.657)	0.018	8.668(1.679–44.756)	0.01
> = 50 kg	57	64.04	17	19.1	89	33.97	1		1	
TB symptoms										
Hemoptysis										
Yes	105	58.01	38	20.99	181	69.08	0.603(0.322–1.129)	0.114	8.928(1.048–76.923)	0.045
No	40	49.38	24	29.63	81	30.92	1		1	
Cavitary										
Yes	42	33.07	42	33.07	127	48.47	5.154(2.71–9.803)	0.000	10.204(2.032–52.631)	0.005
No	103	76.3	20	14.81	135	51.53	1		1	
Treatment										
Standard treatment	136	57.87	53	22.55	235	89.69	2.566(0.966–6.816)	0.059	0.447(0.027–7.449)	0.575
Individualized treatment	9	33.33	9	33.33	27	10.31	1		1	
Hosipitalization										
No	115	58.67	41	20.92	196	74.81	0.509(0.263–0.987)	0.046	0.365(0.066–2.009)	0.247
Yes	30	45.45	21	31.82	66	25.19	1		1	
Liver protection drugs										
Yes	38	48.72	24	30.77	78	29.77	1.779(0.946–3.344)	0.074	1.764(0.935–142.857)	0.056
NO	107	58.15	38	20.65	184	70.23	1		1	
First-line oral anti-TB agents										
Ethambutol										
R	58	54.21	25	23.36	107	40.84	0.987(0.538–1.81)	0.965	1.721(0.37–8.009)	0.489
S	87	56.13	37	23.87	155	59.16	1		1	
Injectable anti-TB agents										
Streptomycin										
R	92	57.5	34	21.25	160	61.07	1.43(0.782–2.614)	0.246	1.85(0.46–7.451)	0.387
S	53	51.96	28	27.45	102	38.93	1		1	
Kanamycin										
R	7	36.84	9	47.37	19	7.25	0.299(0.106–0.843)	0.022	0.137(0.005–3.663)	0.236
S	138	56.79	53	21.81	243	92.75	1		1	
Fluoroquinolones										
Ofloxacin										
R	14	35.9	14	35.9	39	14.89	0.366(0.163–0.825)	0.015	0.793(0.101–6.209)	0.825
S	131	58.74	48	21.52	223	85.11	1		1	
Treatment regularity										
No	2	7.14	18	64.29	28	10.69	29.411(6.535–125)	0.000	47.619(5.025–500)	0.001
Yes	143	61.11	44	18.8	234	89.31	1		1	
Resistant pattern^e^										
R/H	38	52.05	21	28.77	73	27.86	0.693(0.364–1.319)			
R/H/E	13	61.90	4	19.05	21	8.02	1.428(0.447–4.566)			
R/H/S	42	66.67	11	17.46	63	24.05	1.891(0.899–3.978)			
R/H/S/E	37	56.92	12	18.46	65	24.81	1.427(0.686–2.969)			
R/H/Ofx/Km/S/E	3	30.00	6	60.00	10	3.82	0.131(0.026–0.666)	0.014		

^a^others including student and other occupation not investigated in specific

^b^permanent local residence classified into “residence”, otherwise “Floating”

^c^Treatment success including cured(Cure)and treatment completed(TC)

^d^*OR* adjusted odd ratio, *CI* confidence interval

^e^*H* isoniazid, *R* rifampicin, *E* ethambutol, *S* streptomycin, *Km* kanamycin, *Ofx* ofloxacin. Analysis was done for patterns with at least 10 cases in total

Further, a total of 107 (40.84%) of the patients were resistant to ethambutol, 160 (61.07%) resistant to streptomycin, 19 (7.25%) resistant to kanamycin and 39 (14.89%) resistant to ofloxacin based on the DST results. Likewise, treatment outcomes did not indicate any significant difference between these drug-resistant patients (Table 3).

The treatment failure rate in XDR-TB group was 50% (*n* = 9), significantly higher than the Non-XDR-TB group (21.72%, *n* = 53, *P* = 0.006; Table 2). Failure outcomes were more likely to occur if there was resistance to 6 or more drugs (R/H/Ofx/Km/S/E) (*P* = 0.014; Table 2).

In this study, 27 M/XDR-TB patients were treated with individualized regimens according to the DST results and other clinical symptoms. The overall success rate was much lower at 33.33% in comparison to 57.87% for those treated with standard regimen, partly due to that the death rate was much higher (29.63% versus 8.09%). The failure rate was similar between the two treatment regimens (33.33% versus 22.55%).

Further multivariate logistic regression analysis found that treatment failure was associated with baseline weight less than 50 kg (OR, 8.668; 95% CI 1.679–44.756; *P* = 0.010), older than 60 years (OR, 9.053; 95% CI 1.606–51.027; *P* = 0.013), hemoptysis or blood in sputum (OR, 8.928; 95% CI 1.048–76.923; *P* = 0.045), presence of cavitary disease (OR, 10.204; 95% CI 2.032–52.631; *P* = 0.005), or treatment irregularity (OR, 47.619; 95% CI 5.025–500; *P* = 0.001) (Table 3).

Forty (64.52%) of the 62 M/XDR-TB patients failed in the treatment due to adverse effects in the first 10 months of treatment. The median duration from the onset of treatment to fail was 92.5 days (95% CI 123.68–230.98 days). Twenty-two patients in the failure group had also been affected by other factors, such as irregular treatment, poor health condition and other reasons.

Twenty-seven patients (10.31%) died during treatment. Death during treatment was significantly associated with resistance to Ethambutol (*P* = 0.045) or treatment irregularity (*P* = 0.004) (Additional file 1: Table S1).

### Adverse effects

One-hundred-and-twenty two patients (46.55%) experienced major clinically significant adverse effects caused by treatment, of which the most common was gastrointestinal (GI) upset (98, 37.40%), followed by hepatotoxicity (36, 13.74%), arthralgia or muscle pain (25, 9.54%) and allergic reaction (17, 6.49%). There were significantly more cases of allergic reaction in the Non-XDR-TB group (*P* = 0.043). In our study, adverse drug effects occurred frequently in first 3 months of the treatment. The median duration of adverse effects was 92.50 days.

## Discussion

This study first analyzed the treatment outcomes of M/XDR-TB in Zhejiang, China. The overall success rate was 55.34%, with a success rate of 56.97% and 33.33% for patients with MDR-TB (excluding XDR-TB) and XDR-TB, respectively. The overall failure, defaulting and death rates were 23.66%, 6.11% and 10.31%, respectively, with a combined rate of 50.8% contributing to the poor outcome. We identified several risk factors contributing to this poor treatment outcome.

The overall success rate detected in our present study is higher than other reports from China [9, 10]. Liu Q et al*.* [9] reported a success rate of50.7% for patients with MDR-TB and demonstrated that patients with pncA gene mutations, advanced age, and non-standard treatment had a significantly higher risk of poor treatment outcomes. Tang et al*.* [10] reported a success rate of only 40.95% in a cohort of 586 patients with 28.8% being XDR-TB patients. The higher proportion of XDR-TB patients may have contributed to the lower success rate in that study. Reports from other countries showed a success rate ranging from 38.6% to 74.0% [11–14].

A study Carried out in Brazil reported the proportion of unfavourable outcomes was 41.9% among MDR-TB and 81.5% among XDR-TB, which were higher than those in our present study [15]. The study also revealed that bilateral disease, HIV-positive, and comorbidities were associated with death and XDR-TB patients had a 4.7-fold higher odds of an unfavourable outcome [15].

In our present study, of the 140 cured patients, 136 (97%) of the patients converted to smear-negative in the first 6 months treatment course. However, a study conducted by Gao revealed that 24-week bedaquiline treatment combined with personalized anti-TB drug background regimens result in different initial sputum culture conversion rates, which were 84.6% for MDR-TB patients, 83.9% for pre-XDR-TB patients and 86.6% for XDR-TB patients [16]. The findings suggest that completing the initial treatment course of 6 months with injectable agents is critical for treatment success and also implies that any patient not cured during this first 6 months has a high chance of treatment failure and thus requires evaluation at the 6 months for treatment strategies.

In our study, 9 of the 27 M/XDR-TB patients treated with individualized regimens according to the DST results and other clinical symptoms were successful (33.33%) which was less favorable than those used standardized regimens with success rate of 57.87% (Table 3). However, there was no significant difference in the rate of treatment failure between standard and individualized treatments (*P* = 0.575). A study conducted in Korea reported that, delamanid-containing regimens resulted in a higher treatment success rate (81.6%) [17]. Another multi-center study revealed that bedaquiline-containing regimens in the treatment of MDR- and XDR-TB achieved a success of 71.3% (62.4% cured; 8.9% completed treatment).[18] Therefore, together with the introduction of new anti-TB agents, the individualized treatment regimens might be effective to improve MDR-TB treatment outcome.

The primary factors associated with poor treatment outcomes in our study were age (> 60 years), body weight (≤ 50 kg), with cavitary disease or hemoptysis symptoms, and treatment irregularity. In recent study conducted in China indicated that genetic mutation of MTB strains was responsible for some unfavorable outcome [6, 19]. While another study performed in Brazil revealed that bilateral disease, HIV infection and comorbidities were associated with death [13]. These clinical signs are an indication of disease severity and are associated with diagnostic delays and prior long duration of treatment. Treatment irregularity as independent predictors of failure in M/XDR-TB patients was similarly reported by several studies [20, 21] underscoring the importance of patients receiving regular treatment. So the short-course treatment regimens according to drug-sensitivity test were conducted to provide more basis for MDR-TB treatment [22]. Even the study carried out among adolescent achieved treatment success rates of 88% and 83% with the 9-month regimen, and 90% and 75% with the 12-month regimen in adults and children/adolescents [23].

Previous studies suggest an association between resistance to certain anti-TB drugs and poor treatment outcomes in MDR-TB patients [24]. Accumulated studies confirmed that anti-TB-drug resistance always derives from genetic mutations in MTB strains, and MDR-TB was caused by a series of genetic mutations in MTB strains [25, 26]. Our study found no association of resistance to a single drug with treatment failure. However, resistance to 6 or more drugs was associated with treatment failure (*P* = 0.014). The results mean that the more mutations in the MTB strains the more risk of resistances to anti-TB drugs. The molecular mechanisms of MDR-TB incidence indicate that the resistance to anti-TB drugs might be unable to be reversed, so developing new effective anti-TB agents was necessary for further treatment.

Besides drug resistance, adverse effects associated with second-line drugs have been reported as obstacles in the management of M/XDR-TB [27]. In our study, the majority of the 62 M/XDR-TB patients (64.52%) failed treatment were due to adverse effects. This finding advocates that adequate management of adverse effects shall improve treatment outcomes substantially.

The defaulting rate in this study is 6.11%. However, yet another study from China found that defaulting rate is 17% and a higher defaulting rate was also reported from Africa and India [28, 29]. Defaulted M/XDR-TB patients could potentially continue to infect others and are a threat to public health, just as Chisompola et al. reported that primary drug resistance remained the predominant type of transmission [30]. Therefore, reducing defaulting rate is critical to reduce M/XDR-TB spread.

Previous studies in Brazil and South Africa emphasize the roles of unemployment, socially disadvantaged patients, underweight, co-infection with HIV, alcohol and drug abuse and longer treatment duration in association with treatment default [31–34]. China has a massive floating population, and it was reported by Li et al. that the floating population and rural residents were considered high-risk groups for TB infection [35, 36]. This fact makes it especially challenging on treating M/XDR-TB case as risk of treatment default and transfer out is higher and far more difficult to manage in the floating population [35]. Further, treatment of M/XDR-TB is less effective, more toxic and more costly increases the risk of treatment default.

This study has several limitations. First, although a standard protocol and data collection format were used, data on patient’s height and BMI are poorly documented. Second, the number of XDR-TB patients in this study was small. The findings cannot fully represent treatment outcomes of XDR-TB in China. Third, we only had monitoring data for deaths and default in the studied patients. There was no record for the cause of death nor detailed information on default to assess actual reasons. Fourth, this study did not use standardized definitions of adverse effects, their diagnosis or their degree of severity. Despite these limitations, we believe this study provides an important evaluation of treatment outcomes of M/XDR-TB, especially as the first evaluation report in Zhejiang, China of countrywide DOTS-Plus implementation program.

## Conclusion

In conclusion, this study found that the treatment outcome for M/XDR-TB was favorable with a success rate of 55.34% but still not ideal since outcomes for 40.8% patients were poor, consisting of 23.66% failure, 10.31% deaths and 6.11% defaults. Thus substantial effort is required to improve treatment outcomes. Several risk factors identified may be mitigated through clinical management, such as timely diagnosis, regular treatment and controlling adverse effects, and improving social welfare, such as better nutrition and treatment affordability. Our data show that the first 6 months is critical in reducing treatment failure and deaths. This study has important implications for clinical management of M/XDR-TB.

## Supplementary Information

**Additional file 1: Table S1.** Risk factors for death among all M/XDR-TB patients registered for treatment in Zhejiang.

## Data Availability

The supporting data can be acquired via correspondence author.

## Abbreviations

H: Isoniazid
R: Rifampicin
E: Ethambutol
S: Streptomycin
Km: Kanamycin
Ofx: Ofloxacin
