# Supplementary material for: Treatment outcomes of patients with multidrug and extensively drug-resistant tuberculosis in Zhejiang, China
Source: Eur J Med Res. 2021 Apr 3;26:31. doi: 10.1186/s40001-021-00502-0 (PMC8019161; doi:10.1186/s40001-021-00502-0)
Supplement: Supplementary file 1 — Additional file 1: Table S1. Risk factors for death among all M/XDR-TB patients registered for treatment in Zhejiang. [file 40001_2021_502_MOESM1_ESM.docx]

Table S1 Risk factors for death among all M/XDR-TB patients registered for treatment in Zhejiang.

| Chracteristic | Treatment success (cure, TC)^c^ | |  | Death | |  | Total | |  | Univariate analysis | |  | Multivariate analysis | |
| --- | --- | --- | --- | --- | --- | --- | --- | --- | --- | --- | --- | --- | --- | --- |
|  | N=145, n(%) | |  | N=27, n(%) | |  | N=262, n(%) | |  | OR(95% CI)^d^ | *P* |  | OR(95% CI) | *P* |
| Sex |  |  |  |  |  |  |  |  |  |  |  |  |  |  |
| Male | 105 | 54.69 |  | 21 | 10.94 |  | 192 | 73.28 |  | 0.75(0.282-1.994) | 0.564 |  | 0.337(0.015-7.586) | 0.494 |
| Female | 40 | 57.14 |  | 6 | 8.57 |  | 70 | 26.72 |  |  |  |  |  |  |
| Age |  |  |  |  |  |  |  |  |  |  |  |  |  |  |
| <60 year | 124 | 62.94 |  | 14 | 7.11 |  | 197 | 75.19 |  | 5.483(2.262-13.289) | **0.000** |  | 5.282(0.166-168.513) | 0.346 |
| ≥60 year | 21 | 32.31 |  | 13 | 20.00 |  | 65 | 24.81 |  |  |  |  |  |  |
| Occupation^a^ |  |  |  |  |  |  |  |  |  |  |  |  |  |  |
| Farmer | 108 | 54.82 |  | 23 | 11.68 |  | 197 | 75.19 |  | 0.508(0.165-1.564) | 0.238 |  | 1.277(0.081-20.141) | 0.862 |
| Others | 37 | 56.92 |  | 4 | 6.15 |  | 65 | 24.81 |  |  |  |  |  |  |
| Family register^b^ |  |  |  |  |  |  |  |  |  |  |  |  |  |  |
| Resident | 62 | 50 |  | 18 | 14.52 |  | 124 | 47.33 |  | 0.373(0.157-0.887) | **0.026** |  | 0.046(0.001-2.112) | 0.115 |
| Floating | 83 | 60.14 |  | 9 | 6.52 |  | 138 | 52.67 |  |  |  |  |  |  |
| Previous TB treatment |  |  |  |  |  |  |  |  |  |  |  |  |  |  |
| No | 14 | 66.67 |  | 1 | 4.76 |  | 21 | 8.02 |  | 2.779(0.35-22.063) | 0.334 |  | 13.759(0.164-1156.4) | 0.246 |
| Yes | 131 | 54.36 |  | 26 | 10.79 |  | 241 | 91.98 |  |  |  |  |  |  |
| Weight |  |  |  |  |  |  |  |  |  |  |  |  |  |  |
| <50kg | 19 | 44.19 |  | 6 | 13.95 |  | 43 | 16.41 |  | 1.8(0.577-5.614) | 0.311 |  | 1.559(0.077-31.65) | 0.773 |
| >=50kg | 57 | 64.04 |  | 10 | 11.24 |  | 89 | 33.97 |  |  |  |  |  |  |
| TB symptoms |  |  |  |  |  |  |  |  |  |  |  |  |  |  |
| Hemoptysis | | |  |  |  |  |  |  |  |  |  |  |  |  |
| No | 40 | 49.38 |  | 14 | 17.28 |  | 81 | 30.92 |  | 2.827(1.223-6.536) | **0.015** |  | 19.677(0.297-1304.996) | 0.164 |
| Yes | 105 | 58.01 |  | 13 | 7.18 |  | 181 | 69.08 |  |  |  |  |  |  |
| Cavitary |  |  |  |  |  |  |  |  |  |  |  |  |  |  |
| No | 103 | 76.3 |  | 9 | 6.67 |  | 135 | 51.53 |  | 0.204(0.085-0.49) | **0.000** |  | 0.073(0.003-1.624) | 0.098 |
| Yes | 42 | 33.07 |  | 18 | 14.17 |  | 127 | 48.47 |  |  |  |  |  |  |
| Treatment |  |  |  |  |  |  |  |  |  |  |  |  |  |  |
| Standard | 136 | 57.87 |  | 19 | 8.09 |  | 235 | 89.69 |  | 6.363(2.19-18.485) | **0.001** |  | 16.636(0.595-465.474) | 0.098 |
| Individualized | 9 | 33.33 |  | 8 | 29.63 |  | 27 | 10.31 |  |  |  |  |  |  |
| Hosipitalization | |  |  |  |  |  |  |  |  |  |  |  |  |  |
| No | 115 | 58.67 |  | 15 | 7.65 |  | 196 | 74.81 |  | 0.326(0.138-0.77) | **0.011** |  | 1.273(0.101-16.106) | 0.852 |
| Yes | 30 | 45.45 |  | 12 | 18.18 |  | 66 | 25.19 |  |  |  |  |  |  |
| Liver protection drugs | |  |  |  |  |  |  |  |  |  |  |  |  |  |
| NO | 107 | 58.15 |  | 14 | 7.61 |  | 184 | 70.23 |  | 0.382(0.165-0.887) | **0.025** |  | 162.894(0.44-60299.607) | 0.091 |
| Yes | 38 | 48.72 |  | 13 | 16.67 |  | 78 | 29.77 |  |  |  |  |  |  |
| First-line oral anti-TB agents | |  |  |  |  |  |  |  |  |  |  |  |  |  |
| Ethambutol |  |  |  |  |  |  |  |  |  |  |  |  |  |  |
| R | 58 | 54.21 |  | 13 | 12.15 |  | 107 | 40.84 |  | 0.718(0.315-1.638) | 0.431 |  | 0.043(0.002-0.929) | **0.045** |
| S | 87 | 56.13 |  | 14 | 9.03 |  | 155 | 59.16 |  |  |  |  |  |  |
| Injectable anti-TB agents | |  |  |  |  |  |  |  |  |  |  |  |  |  |
| Streptomycin |  |  |  |  |  |  |  |  |  |  |  |  |  |  |
| R | 92 | 57.5 |  | 16 | 10.00 |  | 160 | 61.07 |  | 1.193(0.516-2.761) | 0.680 |  | 0.873(0.049-15.481) | 0.926 |
| S | 53 | 51.96 |  | 11 | 10.78 |  | 102 | 38.93 |  |  |  |  |  |  |
| Kanamycin |  |  |  |  |  |  |  |  |  |  |  |  |  |  |
| R | 7 | 36.84 |  | 2 | 10.53 |  | 19 | 7.25 |  | 0.634(0.124-3.23) | 0.583 |  | 0.968(0.460-1.880) | 0.999 |
| S | 138 | 56.79 |  | 25 | 10.29 |  | 243 | 92.75 |  |  |  |  |  |  |
| Fluoroquinolones | |  |  |  |  |  |  |  |  |  |  |  |  |  |
| Ofloxacin |  |  |  |  |  |  |  |  |  |  |  |  |  |  |
| R | 14 | 35.9 |  | 10 | 25.64 |  | 39 | 14.89 |  | 0.182(0.07-0.473) | **0.000** |  | 0.1(0.003-3.008) | 0.185 |
| S | 131 | 58.74 |  | 17 | 7.62 |  | 223 | 85.11 |  |  |  |  |  |  |
| Treatment regularity | |  |  |  |  |  |  |  |  |  |  |  |  |  |
| Yes | 143 | 61.11 |  | 19 | 8.12 |  | 234 | 89.31 |  | 0.033(0.007-0.168) | **0.000** |  | 0.001(0-0.1) | **0.004** |
| No | 2 | 7.14 |  | 8 | 28.57 |  | 28 | 10.69 |  |  |  |  |  |  |

^a^ others including student and other occupation not investigated in specific.

^b^ permanent local residence classified into “Residence”, otherwise “Floating”

^c^ Treatment success including cured(Cure)and treatment completed(TC)

^d^ OR, adjusted odd ratio; CI, confidence interval.
